# Catalytic mechanism of the colistin resistance protein MCR-1†

**DOI:** 10.1039/d0ob02566f

**Published:** 2021-02-16

**Authors:** Reynier Suardíaz, Emily Lythell, Philip Hinchliffe, Marc van der Kamp, James Spencer, Natalie Fey, Adrian J. Mulholland

**Affiliations:** Centre for Computational Chemistry, School of Chemistry, University of Bristol Cantock's Close Bristol BS8 1TS UK reysuard@ucm.es adrian.mulholland@bristol.ac.uk; School of Biochemistry, University of Bristol, University Walk Bristol BS8 1TD UK; Departamento de Química Física, Facultad de Química, Universidad Complutense 28040 Madrid Spain; School of Cellular and Molecular Medicine, University of Bristol, University Walk Bristol BS8 1TD UK

## Abstract

The mcr-1 gene encodes a membrane-bound Zn^2+^-metalloenzyme, MCR-1, which catalyses phosphoethanolamine transfer onto bacterial lipid A, making bacteria resistant to colistin, a last-resort antibiotic. Mechanistic understanding of this process remains incomplete. Here, we investigate possible catalytic pathways using DFT and *ab initio* calculations on cluster models and identify a complete two-step reaction mechanism. The first step, formation of a covalent phosphointermediate *via* transfer of phosphoethanolamine from a membrane phospholipid donor to the acceptor Thr285, is rate-limiting and proceeds with a single Zn^2+^ ion. The second step, transfer of the phosphoethanolamine group to lipid A, requires an additional Zn^2+^. The calculations suggest the involvement of the Zn^2+^ orbitals directly in the reaction is limited, with the second Zn^2+^ acting to bind incoming lipid A and direct phosphoethanolamine addition. The new level of mechanistic detail obtained here, which distinguishes these enzymes from other phosphotransferases, will aid in the development of inhibitors specific to MCR-1 and related bacterial phosphoethanolamine transferases.

## Abstract 2

Elucidation of the catalytic reaction mechanism of MCR-1 enzyme, responsible for the antimicrobial resistance to colistin, using DFT calculations on cluster models.

## Graphical abstract

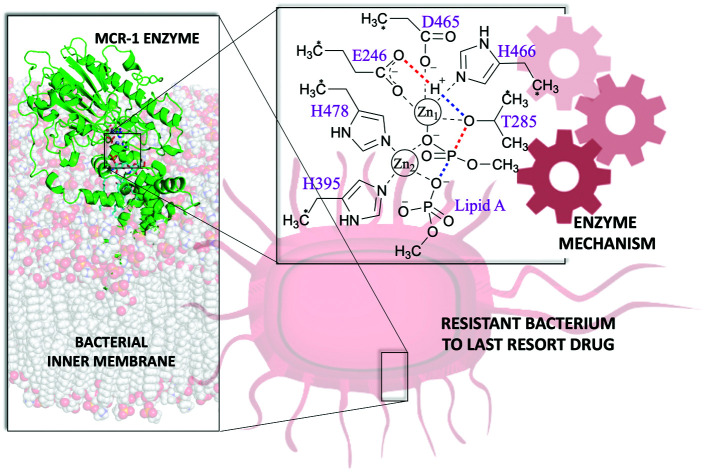

## Introduction

Antimicrobial resistance (AMR) is a major and growing problem in many areas of medicine. AMR has been recognised as one of the greatest threats to human health by the World Economic Forum (WEF).^1^ The polymyxin colistin is currently a ‘last-resort’ antibiotic for extensively-resistant Gram-negative bacteria. Colistin is a positively charged cyclic lipopeptide that is thought to bind to the outer bacterial membrane.^2^ Resistance arises through chemical modification of lipid A, catalysed by enzymes including MCR-1 (mobilized colistin resistance-1) and relatives (such as *Neisseria* EptA),^3^ that reduces binding of the antibiotic. The *mcr-1* gene was identified recently^4^ and is the major cause of colistin failure for *Escherichia coli*, a leading cause of bloodstream infections. Although the structure of the MCR-1 catalytic domain was reported by some of us,^5^ very little experimental information is available regarding the modes of substrate binding and reaction mechanism of this enzyme. It is clear that MCR-1 is an integral, Zn^2+^-dependent inner-membrane protein, with a large periplasmic domain containing the catalytic centre, but the zinc stoichiometry of the system remains uncertain.^5^ Establishing these crucial features should help in the development of inhibitors to counteract colistin resistance.

**Fig. 1:**
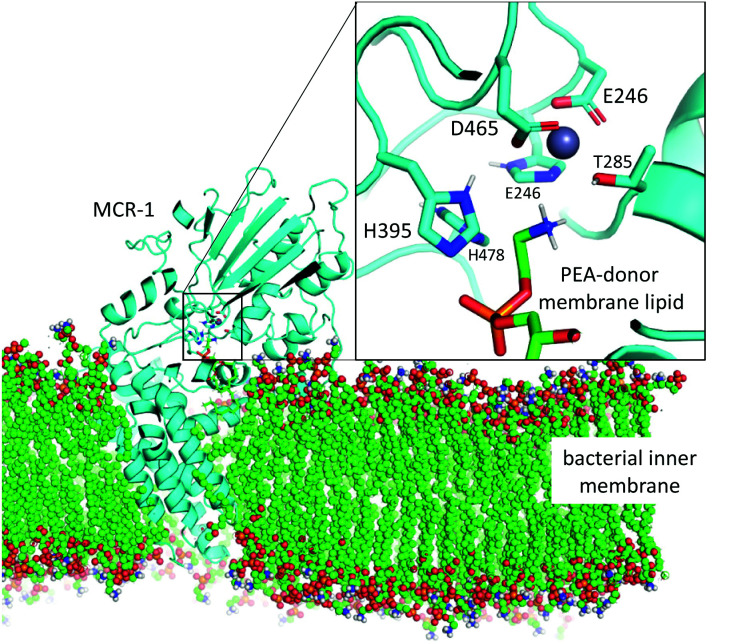
Model of the MCR-1 enzyme (cyan, cartoon) based upon MCR-1 catalytic domain crystal structure^5^ and the full-length *N. meningitidis* EptA structure^40^ shown embedded in bacterial inner membrane (coloured spheres). Mono-Zn active site with PEA-donor phospholipid substrate in coloured sticks. Only polar protons are shown.

Early attempts to simulate phosphoethanolamine (PEA) transfer to the Thr285 acceptor (the first step of the reaction, see Fig. 1) using minimalistic models did not find reasonable reaction barriers and could not explain the role of His395, a residue whose mutation affects colistin susceptibility of MCR-1-expressing bacteria.^5^ In a more recent report, we presented the results of molecular dynamics simulations and preliminary density functional theory (DFT) calculations designed to investigate the feasibility of PEA transfer by the mono- and di-zinc forms of the MCR-1 catalytic domain identified crystallographically.^6^ Those calculations suggested that MCR-1 can support PEA transfer to Thr285 with only one Zn^2+^ ion bound.^6^ Here, we have used a cluster model of the MCR-1 active site to model, for the first time, both steps of the catalytic mechanism using DFT and *ab initio* calculations. Cluster models have been used successfully to study enzyme mechanisms for many years, especially for metallo-proteins.^7–9^ Cluster models are not without limitations, such as non-inclusion of conformational sampling and dynamics, leading to heavy reliance on starting structures, and the use of an implicit solvent model to approximate the cluster's environment that ignores any specific influences. However, we consider that for our purposes cluster models represent an appropriate combination of accuracy and computational efficiency with which to investigate alternative reaction pathways in this relatively complex system. The applications and limitations of cluster models are discussed in detail in many excellent reviews.^7,10^ In this work we address questions about the number of Zn^2+^ ions needed for catalysis, the specific role of these in the reaction, the protonation states of the active site histidine residues 395 and 478 and the structures and energetics of the most probable reaction paths, including barrier heights and the identity of the rate-limiting step. The resulting detailed knowledge of the key electrostatic and structural factors required for catalysis of PEA transfer, as well as identification of reaction pathways, provides information that may be exploited to generate candidate MCR inhibitors. Combining approved drugs with inhibitors of resistance is an established approach to overcoming AMR and prolonging the clinically-useful lifetimes of antibiotics,^11,12^ an important consideration given the continuing weakness of the antibiotic discovery pipeline for Gram-negative bacteria in particular.^13^

## Cluster model and calculations

**Fig. 2:**
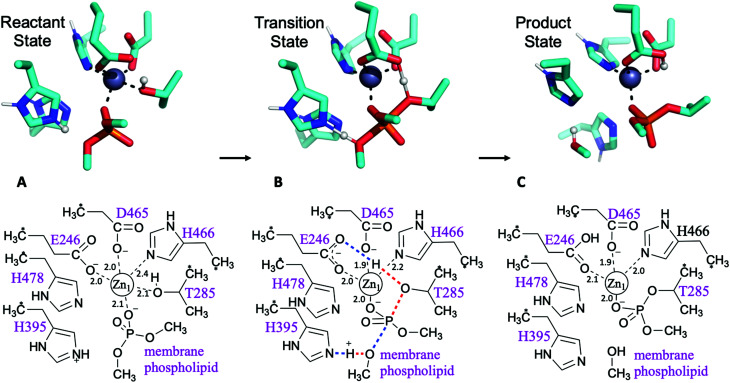
First step of the reaction: phosphoethanolamine transfer to the protein. Stationary points of the proposed reaction pathway are shown in 3D as sticks (top, only selected protons shown, transferring protons in white spheres) and in 2D (bottom). (A) Reactant state. (B) Transition state, concerted transfer of two protons and formation and cleavage of P–O bonds. (C) Product state before substrate departure. Zn-ligand coordination distances indicated in black.

**Fig. 3:**
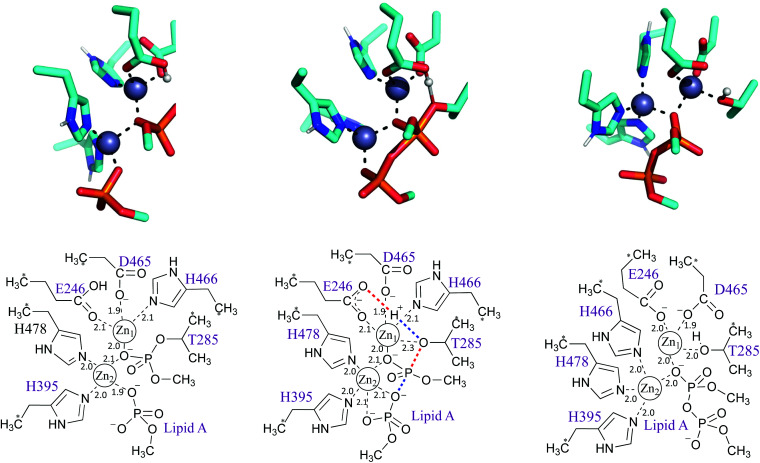
Second step of the reaction: PEA transfer to the lipid A. Stationary points of the proposed reaction pathway are shown in 3D as sticks (top, only selected protons shown, transferring proton in white sphere) and in 2D (bottom). (A) Reactant state after substrate 1 departure and lipid A binding. (B) Transition state, concerted return of proton transferred in the first step and formation/cleavage of P–O bonds. (C) Product state before substrate departure. Zn-ligand coordination distances indicated in black.

Calculations were performed on cluster models derived from X-ray structures determined in previous work, PDB code: 5LRN.^5^ Note that only one zinc site (Zn_1_ in Fig. 2 and 3), tetrahedrally coordinated by MCR-1 residues Glu246, His466, Asp465 and Thr285, is conserved in most PEA transferases.^5^ This is the Zn^2+^ ion position (Zn^2+^_1_) used hereafter when referring to a mono-zinc structure. Substrates for the first (phosphatidylethanolamine, PEA) and second (lipid A) steps of the reaction were modelled by deprotonated dimethyl- and methyl-phosphate molecules respectively (see Fig. 2 and ESI†). In the case of the two-Zn^2+^ ion system, the initial position of the second Zn^2+^ ion (Zn_2_ in Fig. 3) was taken from the di-zinc MCR-1 crystal structure (PDB code: 5LRM).^5^ All Cα atoms were kept frozen at their corresponding positions in the X-ray crystal structure during the calculations to preserve the approximate spatial arrangement of the residues. The cluster model for PEA transfer to the protein (step 1 of the reaction) consisted of 95 atoms and the cluster model for PEA transfer to lipid A (step 2 of the reaction) consisted of 99 atoms. The total charge of the models takes the value 0, ±1 depending upon the protonation states of the histidine residues.

Geometry optimizations were performed using the B3LYP-GD3BJ method (standard B3LYP functional^14^ with the D3 version of Grimme's dispersion correction with Becke–Johnson damping)^15^ as implemented in the Gaussian 09 package.^16^ Inclusion of dispersion is important in modelling enzyme-catalysed reactions with DFT.^17^ A combination of the 6-31+G(d,p) basis set for the phosphorus and the oxygen atoms coordinated to Zn^2+^, the SDD Stuttgart/Dresden effective core potential for Zn, and the 6-31G(d) basis set for all other atoms, was used. This combination of functional and basis set has proved to deliver satisfactory results when modelling enzyme reactions,^18–20^ but in order to test the accuracy of the DFT results, additional *ab initio* single point calculations at the RI-SCS-MP2 level and a larger basis set aug-cc-pVTZ were performed in Orca v4.2.3.^21,22^ SCS-MP2^23^ has been shown to give more accurate results than pure MP2 for (enzyme) reaction barriers and energies.^24–27^ Solvation effects were taken into account by the use of the conductor-like polarized continuum model (C-PCM)^28^ and a dielectric constant, *ε* = 4, as widely used in DFT cluster model calculations of enzymes.^7,8^ It was previously demonstrated that the first step of the reaction was not sensitive to the exact *ε* value used.^6^ Here, we show that the same applies for the second step of the reaction (Table S4†). Since typically the effect of *ε* on DFT cluster model calculations saturates with increasing system size,^29^ we consider the relatively small cluster used here to be appropriate for this system. Frequency calculations were performed at the same level of theory as the geometry optimizations to obtain free energy corrections (at 298.15 K and 1 atm pressure) and to confirm the nature of the stationary points. Due to use of frozen atoms in the model, some imaginary frequencies occur at the stationary points, but they are small and confined to the vicinity of the frozen atoms. Our discussion focuses on the ‘best method’ data calculated, but a full breakdown of energies can be found in the ESI.†

## Phosphoethanolamine transfer to the protein

Possible mechanisms for PEA transfer to the Thr285 acceptor, the first step of the two-step reaction mechanism, have previously been studied using DFT cluster model calculations.^5,6^ Here we explored an expanded range of alternative mechanisms, assessed these using higher levels of theory, and extended the study to the whole reaction. Exploratory studies of the different potential reaction pathways and different choices of protonation states for the histidine residues were carried out using semiempirical PM6 Hamiltonian and B3LYP/6-31G(d) levels of theory before being submitted to B3LYP-GD3BJ calculations and the previously described combination of basis sets (see ESI for a detailed description and Table S1† for results). The orientation of the incoming substrates in the model was based on the likely orientation of MCR-1 in the membrane (Fig. 1 and ESI, section S2†).

Pathways assessed included (but were not limited to): a. shuttling of a proton to the leaving group by the transient phosphoryl group; b. cleavage of the phosphate group concerted with proton transfers from Thr285 to Glu246 and His395 to the leaving group; c. the same pathway with His478 protonated; and d. a two Zn^2+^ ion-mechanism with Thr285 deprotonated by Glu246 and the leaving group stabilised by the second metal ion. Other attempts involving a non-protonated leaving group were also considered and discarded. Most pathways tested were discarded due to failure to find a transition state, although preliminary estimations pointed to much higher barriers. All viable and complete pathways are shown in Table S1.† We then confirmed, on the basis of comparing free energies and barrier heights, that the most likely reaction path for PEA transfer from the bacterial lipid membrane to the protein involves nucleophilic attack of Thr285 on the phosphate centre of the phospholipid, concerted with activation of Thr285 by Glu246 and a proton transfer from His395 to the dephosphorylated lipidic leaving group (Fig. 2). This phosphate cleavage concerted with proton transfer is evidenced by the changes in geometry from reactants to products reflected in the normal mode of the transition state imaginary frequency (see animation in ESI†). Concerted reaction paths have been found to be lower in energy than the corresponding stepwise paths observed in other phosphate-processing enzymes.^30–32^ The same concerted reaction path is found if both histidine residues 395 and 478 are protonated, with a potential energy reaction barrier height of 12.6 kcal mol^−1^ at the SCS-MP2 level (comparing favourably with the barrier of 19.1 kcal mol^−1^ obtained when only His395 is protonated) and an energy of reaction of −8.8 kcal mol^−1^ (Table S1†). When taking into account the free energy and solvent corrections from DFT calculations, this gave a free energy reaction barrier of 16.9 kcal mol^−1^ with a corresponding −8.0 kcal mol^−1^ free energy of reaction (Fig. 2 and Table 1). As the p*K*_a_ of the conjugate acid of imidazole is approximately 7,^33^ small shifts in pH could easily change the protonation state of histidine side chains. Proton affinity calculations were thus performed and showed a preference for histidine residues 395 and 478 to be protonated. This preference increases in the presence of the phosphorylated substrate, see Table S2† for details. As previously reported,^5^ mutation of His395 to Ala completely destroys the activity of the enzyme, suggesting a direct involvement of this residue in the reaction mechanism, consistent with our proposed reaction pathway.

**Table 1:** Calculated barrier heights (Δ*G*^‡^) and reaction energies (Δ*G*, kcal mol^−1^) for different numbers of Zn^2+^ ions and different reaction pathways. SCS-RI-MP2/aug-cc-pVTZ//B3LYP-GD3BJ/B1^a^

	One Zn^2+^	Two Zn^2+^
1^st^ step		
Δ*G*^‡^	16.9	39.6
Δ*G*	−8.0	26.2
2^nd^ step		
Δ*G*^‡^	Not found	12.0
Δ*G*		−10.5

a SCS-RI-MP2 single point calculations on geometries optimised at DFT level of theory, taking into account the free energy and solvent corrections from DFT calculations, see Table S4.† B1 = 6-31+G(d,p) for the P and the O atoms coordinated to Zn, the SDD Stuttgart/Dresden ECP for Zn, and the 6-31G(d) basis set for all other atoms.

The same procedure was followed for the two-Zn^2+^ system. A transition state structure was found, indicating a similar pathway to that observed in the one-Zn^2+^ system. However, we could not identify a reactant state connected to this transition state compatible with the crystal structure: any effort to locate it led to a geometry in which His466 changed from coordinating the primary metal ion (Zn^2+^_1_) to coordinating the second zinc (Zn^2+^_2_). The values reported in Table 1 correspond to this system, with a reactant state not matching the crystal structure. On the other hand, the single point energy of a hypothetical reactant state geometry where His466 stays coordinated to was calculated. The resulting reaction barrier for PEA transfer to Thr285 implies that the second Zn^2+^ ion is not required for the first step of the reaction mechanism since it can proceed with a single Zn^2+^ ion.

## Phosphoethanolamine transfer to the lipid A

The second step of the reaction is assumed to be the nucleophilic attack of one of the phosphate head groups attached to lipid A on the phosphoryl group of PEA attached to Thr285. To model this process, various reaction pathways equivalent to those tested for the first step were assessed, and most discarded as we were unable to find a TS structure for the system with one Zn^2+^ ion. In contrast, it was easy to find all stationary points for the system when two Zn^2+^ ions were present (Fig. 3). For the reactant state, the first Zn^2+^ ion (Zn^2+^_1_) is tightly coordinated to one oxygen atom of the phosphoryl group attached to Thr285 (distance of 2.0 Å) and the side chain oxygen of Thr (Oγ) is detached from the cation at a distance of 3.0 Å, see Fig. 3A. The second Zn^2+^ ion (Zn^2+^_2_) holds the incoming phosphate group of lipid A and guides it to the phosphate group attached to Thr285. In the reactant state, lipid A coordinates Zn^2+^_2_ through one oxygen of the phosphate group (the prospective nucleophile); coordination strengthens in the TS with addition of a second oxygen and the nucleophilic oxygen retaining interaction at a distance of 2.1 Å, see Fig. 3B. Both Zn^2+^ ions show favourable tetrahedral coordination through all the stationary points of the reaction. The transition state involves proton transfer from Glu246 (protonated in the first step of the reaction) to Thr285 Oγ, concerted with phosphate release from Thr285 *via* cleavage of the bond between Oγ and the P atom (Fig. 3B). This second step is exothermic (−10.5 kcal mol^−1^) and faster than the previous step (free energy reaction barrier of 12.0 kcal mol^−1^) according to the SCS-RI-MP2 calculations with solvent and free energy corrections, see Table 1, S3 and S4.† In the product state, the transferred phosphoryl group is coordinated to both Zn^2+^ ions *via* a single, bridging oxygen atom. Coordination of by Thr285 is restored, displacing His466, which coordinates the ion at the reactant and transition states.

Following the transition state, His466 now coordinates Zn^2+^_2_, which is probably required to obtain the product complex, after phosphoryl transfer and restoration of the Thr285:Zn^2+^_1_ interaction. We speculate that this additional coordination of Zn^2+^_2_ may facilitate the release of the modified lipid A (also coordinated to Zn^2+^_2_; Fig. 3C). Subsequently, restoration of the enzyme to its resting state would involve release of Zn^2+^_2_ and restoration of Zn^2+^_1_ coordination by His466. It should be noted, however, that the cluster model employed in this work does not consider the possibility of any residue from the transmembrane domain (TMD) coordinating Zn^2+^_2_. As, based on comparisons with *N. meningitidis* EptA, the Zn^2+^_2_ site may lie close to the TMD in the intact, full-length, enzyme, any involvement of the TMD in coordinating this ion could preclude this proposed motion of His466. However, as at present crystal structures are only available for the MCR-1 periplasmic catalytic domain, additional structural work is necessary before this point can be clarified.

## Role of the Zn^2+^ ions in the rate-determining step

Comparison of calculated free energy barriers for the two reaction steps (Table 1) indicates that the first of these (PEA transfer to the protein) is likely to be rate-determining. To analyse the role of the Zn^2+^ ion(s) in the rate-limiting step, further single point calculations on the stationary points were performed at the DFT level, see ESI for details.† Here, the Zn^2+^ ion was replaced by a +2 point charge and the energy recalculated without any change in the geometry. The energy difference between the transition and reactant states does not increase (as would be expected if the Zn^2+^ orbitals are directly involved in the chemical reaction), but instead decreases (see Table S6†). This result suggests that the Zn^2+^ does not have a direct involvement in the reaction, but its function is simply to hold the reactants in place and to provide electrostatic stabilisation to the TS. In this case, the reaction rate would be expected to be insensitive to the identity of the metal ion present, *i.e.*, calculations with different metal ions would be expected to give similar barrier heights. This is the case when Zn^2+^ is replaced with Mg^2+^ and even Na^+^ (see Table S6†), in DFT calculations that do not allow for structural changes. If the geometry is allowed to change, the barrier height is still very similar for Mg^2+^ and increases somewhat for Na^+^ compared to the value obtained without geometry optimization (Table S6†). Taken together, these calculations point to the hypothesis that Zn^2+^ orbitals are not directly involved in the reaction.^34^

## Conclusions

We identify a complete reaction mechanism for MCR-1 from QM calculations on active site models. The first step, direct transfer of PEA from a membrane phospholipid to Thr285, involves phosphate cleavage concerted with two proton transfers: one from Thr285 to the carboxylate of Glu246 and another from His395 to the leaving group. This is the rate-limiting step and can proceed with a single Zn^2+^ ion. This Zn^2+^ ion is important for structural organisation of the active site with the bound substrate and presumably for transition state stabilisation, but the involvement of its orbitals in the chemical reaction is limited. In contrast to the first step, transfer of PEA to lipid A cannot proceed without a second Zn^2+^ metal ion, implying that this must be recruited either directly to the covalent Thr285-phospho-intermediate or during lipid A binding. Recruitment of a second zinc ion (Zn^2+^_2_) could occur either directly to the Zn_2_ site after PEA addition to Thr285 or involve incoming lipid A arriving with zinc already attached to the acceptor phosphate group. In addition, deprotonated His395, generated after the first step of the reaction, may also contribute to binding, consistent with recent proposals regarding the role of histidine residues as cation recruiters in phosphate processing enzyme systems.^35^ This step is predicted to have a lower barrier and to be more exothermic than the first step of the reaction mechanism. This proposed “ping-pong” reaction mechanism shares similarities with those of alkaline phosphatase enzymes,^36,37^ see ESI† for details, but is distinguished from these by the ability to transfer PEA from a membrane phospholipid to Thr285 using a single, rather than two, Zn^2+^ ion.

By identifying species along the reaction pathway and establishing the contributions of specific active site residues to the MCR catalytic mechanism, we here provide detailed mechanistic proposals with implications for future development of inhibitors for MCR and related enzymes. Co-administration with inhibitors of resistance represents a validated strategy to extend the therapeutically useful lifetime of antibiotics.^38^ In this instance, our findings suggest that approaches that hinder metal ion access to a second zinc site represent one possible route to MCR inhibition.^39^

## Conflicts of interest

The authors have no conflicts of interest to declare.

## Supplementary Material

OB-019-D0OB02566F-s001

OB-019-D0OB02566F-s002

OB-019-D0OB02566F-s003
