# Supplementary material for: Catalytic mechanism of the colistin resistance protein MCR-1
Source: Org Biomol Chem. 2021 Feb 16;19(17):3813–9. doi: 10.1039/d0ob02566f (PMC8097703; doi:10.1039/d0ob02566f)
Supplement: OB-019-D0OB02566F-s002 [file OB-019-D0OB02566F-s002.docx]

Supporting Information

Catalytic Mechanism of the Colistin Resistance Protein MCR-1

Reynier Suardíaz,^a,b,c*^ Emily Lythell,^a,d^ Philip Hinchliffed,^d^ Marc van der Kamp,^a,b^ James Spencer,^d^ Natalie Fey,^a^ Adrian J. Mulholland ^a*^

1. Centre for Computational Chemistry, School of Chemistry, University of Bristol, Cantock’s Close, Bristol BS8 1TS, UK.
2. School of Biochemistry, University of Bristol, University Walk, Bristol BS8 1TD, UK.
3. Departamento de Química Física, Facultad de Química, Universidad Complutense, 28040 Madrid, Spain.
4. School of Cellular and Molecular Medicine, University of Bristol, University Walk, Bristol BS8 1TD, UK.

^*^ [reysuard@ucm.es](mailto:reysuard@ucm.es); [Adrian.Mulholland@bristol.ac.uk](mailto:Adrian.Mulholland@bristol.ac.uk)

Table of Contents

[S1- Computational details of the DFT and ab initio calculations 2](#_Toc63361921)

[S2- Orientation of the (membrane) substrate 3](#_Toc63361922)

[S3- Reaction pathways for phosphoethanolamine transfer to Thr285 (first step of the reaction) 4](#_Toc63361923)

[S4- Protonation state of H395 and H478 5](#_Toc63361924)

[S5- Reaction pathways for phosphoethanolamine transfer to lipid A (second step of the reaction) 6](#_Toc63361925)

[S6- Ab intio SCS-MP2 energies for the lowest energy reaction path 6](#_Toc63361926)

[S-7 Sensitivity of the second step of the reaction to ε 7](#_Toc63361927)

[S8- Role of the Zn2+ ion in the rate-limiting step 7](#_Toc63361928)

[S9- Comparison between MCR and Alkaline Phosphatase 7](#_Toc63361929)

[Coordinates 8](#_Toc63361930)

[References 8](#_Toc63361931)

# S1- Computational details of the DFT and ab initio calculations

Model building: A cluster model was derived from X-ray structure determined in previous work, PDB code: 5LRN.^1^ This structure has only one Zn^2+^ ion in the active site. Therefore, for the two-Zn^2+^ ion structures, the position of the second ion was taken from the di-zinc MCR-1 crystal structure (PDB code: 5LRM).^1^ All Cα atoms were kept frozen at their corresponding positions in the X-ray crystal structure during the calculations to preserve the approximate spatial arrangement of the residues.

DFT calculations: All DFT calculations were performed with the Gaussian 09 package.^2^ Preliminary exploratory studies were carried out with PM6 semiempirical method where calculated reaction barriers were suspiciously low, see Table S1. We then switched to DFT level of theory and started from scratch. B3LYP functional was then used with a combination of the 6-31+G(d,p) basis set for the phosphorus and the oxygen atoms coordinated to Zn, the SDD Stuttgart/Dresden effective core potential for Zn, and the 6-31G(d) basis set for all other atoms. The same functional and basis set combination but with Grimme’s dispersion correction with Becke-Johnson damping^3^ was used for the definitive geometry optimizations of all the stationary points along the reaction paths. Solvation effects were taken into account at the same level of theory by the use of the conductor-like polarized continuum model (C-PCM)^4^ and ε = 4, as widely used in DFT cluster model calculations of enzymes.^5, 6^ Frequency calculations were performed at the same level of theory as the geometry optimizations to obtain free energy corrections at 298.15 K and 1 atm pressure as well as to confirm the nature of the stationary points. Due to the frozen atoms in the model, some small imaginary frequencies occur at the stationary points, but they are very small (< 30i cm^-1^) and confined to the vicinity of the frozen atoms. If we consider only zero-point corrections to avoid potential inaccuracies in the calculated harmonic entropy, results are very similar and do not affect any of the conclusions.

*Ab initio* calculations: Additional single point calculations at the SCS-MP2 level and a larger basis set aug-cc-pVTZ for all the atoms, using the Resolution of Identity (RI) approximation on Coulomb integrals and exchange integrals, were performed with Orca v4.2.3^7, 8^ software over the DFT-obtained geometries. Corrections for the free energy and solvent effect were added based on the B3LYP-D3 previous calculations.

Choices of reaction paths: Different reaction paths for PEA transfer to the MCR Thr285 acceptor were considered. Some of them were just discarded due to failure to find a transition state. The final candidates are shown in Table S1 and consist of: a. shuttling of a proton to the leaving group by the transient phosphoryl group; b. cleavage of the phosphate group concerted with proton transfers from Thr285 to Glu246 and His395 to the leaving group; c. the same pathway with His478 protonated; and d. a two Zn^2+^ ion-mechanism with Thr285 deprotonated by Glu246 and the leaving group stabilized by the second metal ion.

.

# S2- Orientation of the (membrane) substrate

A BLAST^9^ search of the full-length *Escherichia coli* MCR-1 sequence (membrane and extracellular domains; UniProt A0A0R6L508) against the full Protein Data Bank^10^ reveals significant identity (37%) with the lipid A phosphoethanolamine transferase from *Neisseria meningitidis* (PDB code: 5FGN^11^). Membrane orientation was retrieved for 5FGN from the Orientations of Proteins in Membranes (OPM) database.^12^ Structural alignment of a previously obtained crystal structure for the MCR-1 catalytic domain (PDB code: 5LRN^1^) yields the relative orientation of MCR-1 towards the membrane (Figure S1) and hence the likely direction of substrate entry into the active site. In this way, we were able to confirm that the orientation of the phospholipid substrate in the subsequent calculations is sensible.

|  |
| --- |
| Figure S1. Structural alignment of MCR-1 protein (PDB code 5LRN) to EptA from *Neisseria meningitidis* showing the relative orientation of bacterial membrane and approximate orientation of phospholipid MCR-1 substrate. |

# S3- Reaction pathways for phosphoethanolamine transfer to Thr285 (first step of the reaction)

First, exploratory studies were carried out using semiempirical PM6 and later, further calculations were performed using DFT, with both B3LYP and B3LYP-GD3BJ, as described above. Different alternative pathways were tested and discarded. Here we show only those pathways where transition states were found. Single point calculations using SCS-RI-MP2 and the aug-cc-pVTZ basis set were performed on the DFT-optimized geometries.

| Table S1. Pathways considered for the first step of the reaction mechanism and the corresponding potential and free energies of activation and reaction in kcal mol^-1^ at different levels of theory. Solvent and free energy contributions at B3LYP-D3BJ level. A) a proton is transferred to the leaving group, shuttled by the transient phosphoryl group. B) concerted pathway where the phosphate group is cleaved at the same time that the proton of Thr285 is transferred to Glu246 and one proton from His395 is transferred to the leaving group, H478 neutral. C) similar to B but H478 protonated D) similar to B, C but using two Zn^2+^ ions and H395/478 neutral. | | | | | | |
| --- | --- | --- | --- | --- | --- | --- |
|  |  | Δ^‡^E | ΔE | Δ^‡^G | ΔG | 2D representation |
| A | PM6 | 5.2 | -3.7 | -9.1 | -4.5 |  |
|  | B3LYP | 36.3 | -2.4 | 37.3 | -2.1 |  |
|  | B3LYP- GD3BJ | 38.1 | 4.5 | 41.2 | 8.7 |  |
|  | B3LYP- GD3BJ (e=4) | 31.8 | 2.6 | -- | -- |  |
|  | Solvent contribution* | -6.3 | -1.9 | -- | -- |  |
|  | Free Energy contribution* | -- | -- | 3.1 | 4.2 |  |
|  | SCS-MP2 | 34.1 | 14.1 | -- | -- |  |
|  | SCS-MP2 + contributions | -- | -- | 30.9 | 16.4 |  |
| B | PM6 | 3.3 | -0.8 | 0.6 | -6.9 |  |
|  | B3LYP | 16.0 | -7.9 | 12.9 | -12.0 |  |
|  | B3LYP- GD3BJ | 15.7 | -4.2 | 20.9 | -5.0 |  |
|  | B3LYP- GD3BJ (e=4) | 22.5 | 7.4 | -- | -- |  |
|  | Solvent  contribution* | 6.8 | 11.6 | -- | -- |  |
|  | Free energy  contribution* | -- | -- | 5.2 | -0.8 |  |
|  | SCS-MP2 | 19.1 | -3.5 | -- | -- |  |
|  | SCS-MP2 + contributions | -- | -- | 31.1 | 7.3 |  |
| C | PM6 | 0.5 | -6.8 | -1.7 | -15.5 |  |
|  | B3LYP | 29.8 | -3.1 | 12.5 | -29.4 |  |
|  | B3LYP- GD3BJ | 13.0 | -5.1 | 10.2 | -11.5 |  |
|  | B3LYP- GD3BJ (e=4) | 20.1 | 2.2 | -- | -- |  |
|  | Solvent  contribution* | 7.1 | 7.3 | -- | -- |  |
|  | Free energy  contribution* | -- | -- | -2.8 | -6.4 |  |
|  | SCS-MP2 | 12.6 | -8.8 | -- | -- |  |
|  | SCS-MP2 + contributions | -- | -- | 16.9 | -8.0 |  |
| D | PM6 | TS not found | | | |  |
|  | B3LYP | 36.8 | 16.3 | 30.3 | 12.2 |  |
|  | B3LYP- GD3BJ | 37.3 | 19.9 | 33.5 | 17.0 |  |
|  | B3LYP- GD3BJ (e=4) | 34.6 | 20.2 | -- | -- |  |
|  | Solvent  contribution* | -2.7 | 0.3 | -- | -- |  |
|  | Free Energy contribution* | -- | -- | -3.8 | -2.9 |  |
|  | SCS-MP2 | 37.2 | 20.8 |  |  |  |
|  | SCS-MP2 + contributions | -- | -- | 30.7 | 18.3 |  |
| ^*^ Solvent corrections based on the difference between column B3LYP- (ε=4) and B3LYP-GD3BJ . Free Energy corrections based on the difference between B3LYP-D3 ΔG and ΔE values. | | | | | | |

As can be seen in Table S1, a concerted pathway, where the phosphate group is cleaved at the same time that the proton of Thr285 is transferred to Glu246 and one proton from histidine 395 is transferred to the leaving group, is plausible according to the reaction activation energies (pathways B and C). As His395 is donating a proton, this residue should be protonated. The same pathway, but using two Zn^2+^ ions, is too slow for enzymatic performance, with a predicted reaction barrier over 30 kcal/mol (pathway D).

# S4- Protonation state of H395 and H478

As mentioned before, at least H395 should be protonated since it is participating in the phosphoethanolamine transfer by donating a proton to the leaving group. We have estimated the relative proton affinity of the cluster model at different protonation states of the histidine residues 395 and 478. As can be seen in Table S2, the protonated states of both histidine residues are preferred according to all the calculations and this preference increases in the presence of the substrate. These proton affinities should be taken as approximate, able to show only a tendency or preference for the protonation state and not a usable value for proton affinity.

To estimate the relative proton affinities, we calculated the energy difference between the neutral and charged forms and balanced the system with the change of pronation of the solvent. Therefore, we calculated the energy for a system as shown in equation 3 ,where the solvent molecule does not interact with the model-His system and the energy of the water molecules is calculated at the same level of theory as the cluster model calculations.

Model-His-neutral + H^+^ => Model-His-charged (1)

H_3_O^+^ => H_2_O + H^+^ (2)

----------------------------------------------------------------------------------------------------------------

Model-His-neutral + H_3_O^+^ => Model-His-charged + H_2_O (3)

| Table S2 Relative proton affinities (equation 3) of different protonation states of H395 and H478 (kcal/mol). | | | | | | | |
| --- | --- | --- | --- | --- | --- | --- | --- |
| H395 | H395 | H478 | H478 |  |  |  |  |
| H in δ2 | H in ε1 | H in δ2 | H in ε1 | vacuum | ε=4 | vacuum, no substrate | ε=4, no substrate |
| x |  | x |  | 263.0 | 78.6 | 106.2 | --^*^ |
|  | x |  | x | 238.1 | 75.2 | 110.5 | 48.4 |
| x |  |  | x | 240.5 | 76.0 | 109.5 | 46.8 |
|  | x | x |  | 238.7 | 72.9 | 106.6 | 46.8 |
| x | x | x | x | 0.0 | 0.0 | 0.0 | 0.0 |
| x |  | x | x | 90.7 | 32.0 | 22.9 | 16.4 |
|  | x | x | x | 86.3 | 28.6 | 18.4 | 16.6 |
| x | x | x |  | 84.7 | --^*^ | 22.4 | 15.8 |
| x | x |  | x | 89.9 | 30.7 | 19.5 | 15.1 |
| ^*^ -- means that proton affinities were not determined due to problems with converging the geometry optimization. These not-converged geometries are not used in the reaction path calculations. | | | | | | | |

These results show a preference for both histidine residues to be protonated. This preference increases in the presence of the phosphorylated substrate. It is known that mutation of MCR-1 His395 to Ala completely disrupts the activity of the enzyme,^1^ suggesting a direct involvement of this residue in the reaction mechanism, which is consistent with the proposed reaction pathway.

# S5- Reaction pathways for phosphoethanolamine transfer to lipid A (second step of the reaction)

The second step of the reaction is assumed to be the nucleophilic attack of the phosphate head of lipid A to the phosphoryl group now attached to Thr285. We were unable to find a TS at DFT level with a one-Zn system, but it was very easy to find all stationary points for the system with two Zn^2+^ ion, see Table S3.

| Table S3. Considered pathways for the second step of the reaction mechanism and the corresponding potential and free energies of activation and reaction in kcal/mol. A) Concerted pathway where the phosphate group is cleaved at the same time that the proton of Thr285 is transferred to Glu246 with one Zn^2+^. B) similar to A but using two Zn^2+^ ions. | | | | | | | | | |
| --- | --- | --- | --- | --- | --- | --- | --- | --- | --- |
|  | A | | B | | | | | | |
|  |  | |  | | | | | | |
|  | PM6 | B3LYP and B3LYP-D3 | PM6 | B3LYP | B3LYP-D3 | B3LYP-D3 (ε=4) | Solvent contribution* | Free energy contribution* | SCS-MP2 |
| ΔE^‡^ | 20.7 | TS not found | TS not found | 6.7 | 2.1 | 9.4 | 7.2 | -- | 3.3 |
| ΔE | 15.9 |  |  | -28.5 | -34.1 | -21.7 | 12.4 | -- | -23.9 |
| ΔG^‡^ | 18.62 |  |  | 9.6 | 3.5 | -- | -- | 1.4 |  |
| ΔG | 17.22 |  |  | -26.5 | -33.1 | -- | -- | 1.0 |  |
| ^*^ Solvent corrections based on the difference between column B3LYP-D3 (ε=4) and B3LYP-D3. Free Energy corrections based on the difference between B3LYP-D3 ΔG and ΔE values. | | | | | | | | | |

# S6- Ab intio SCS-MP2 energies for the lowest energy reaction path

Summary of the SCS-MP2 energies with the corrections based on DFT calculations for the lowest energy reaction path.

| Table S4. Calculated barrier heights and reaction energies (kcal mol^–1^) for different number of Zn^2+^ ions for the lowest energy reaction paths in the two steps f the reaction. | | | | | |
| --- | --- | --- | --- | --- | --- |
|  |  | 1^st^ step | | 2^nd^ step | |
|  |  | activation energy | reaction energy | activation energy | reaction energy |
| One Zn^2+^ | SCS-MP2 ΔE | 12.6 | -8.8 | not found | |
|  | Free energy contribution^*^ | -2.8 | -6.4 |  |  |
|  | Solvent  contribution^*^ | 7.1 | 7.3 |  |  |
|  | SCS-MP2 ΔG | 16.9 | -8.0 |  |  |
| Two Zn^2+^ | SCS-MP2 ΔE | 37.2 | 20.8 | 3.3 | -23.9 |
|  | Free energy contribution^*^ | -3.8 | -2.9 | 1.4 | 1.0 |
|  | Solvent  contribution^*^ | -2.7 | 0.3 | 7.2 | 12.4 |
|  | SCS-MP2 ΔG | 30.7 | 18.3 | 11.9 | -10.5 |
| ^*^Solvent corrections based on the difference between B3LYP-D3 (C-PCM, ε=4) and B3LYP-GD3BJ energies. Free Energy corrections based on B3LYP-D3 frequency calculations. | | | | | |

# S-7 Sensitivity of the second step of the reaction to ε

In previous work,^1^ sensitivity to the dielectric constant ε of reaction barrier and energy was demonstrated to be low for the first step of the reaction. Here we have used the stationary point geometries to carry out single point calculations at different dielectric constants to check the sensitivity of the reaction barrier height and energy of reaction of the second step of the reaction to this property. Variations below 2 kcal mol^-1^, which may be considered rather small, were found when scanning ε from 3 to 15, see Table S4. Since typically the effect of ε on DFT cluster model calculations saturates with the size of the system,^13^ we consider the relatively small cluster used here to be appropriate in size.

| Table S5. Potential energies of activation and reaction obtained by single point calculations at B3LYP-D3 level for the first step of the reaction. | | | | | | | | | | | | | |
| --- | --- | --- | --- | --- | --- | --- | --- | --- | --- | --- | --- | --- | --- |
|  | ε=3 | ε=4 | ε=5 | ε=6 | ε=7 | ε=8 | ε=9 | ε=10 | ε=11 | ε=12 | ε=13 | ε=14 | ε=15 |
| ΔE^‡^ | 22.7 | 23.4 | 23.8 | 24.2 | 24.4 | 24.6 | 24.7 | 24.9 | 25.0 | 25.0 | 25.1 | 25.2 | 25.2 |
| ΔE | 3.9 | 4.7 | 5.2 | 5.6 | 5.8 | 6.0 | 6.2 | 6.3 | 6.4 | 6.5 | 6.6 | 8.5 | 6.7 |

# S8- Role of the Zn2+ ion in the rate-limiting step

The Zn^2+^ ion was replaced by Mg^2+^, Na^+^ and a 2+ point charge in cluster model calculations of the first, rate-limiting, step of the reaction. Single point calculations (without any change in the geometry) and geometry optimization were performed at DFT level. The resulting values of reaction barrier heights suggest that Zn^2+^ orbitals do not have a direct involvement in the reaction. The level of theory used was the same as used before: B3LYP-GD3BJ functional with the 6-31+G(d,p) basis set for phosphorus and oxygens coordinated to Zn, SDD Stuttgart/Dresden effective core potential for Zn and 6-31G(d) basis set for all other atoms.

| Table S6. Reaction barrier heights and reaction energies (kcal/mol) when replaced Zn^2+^ ion. sp=single point and opt=geometry optimization | | | | | | |
| --- | --- | --- | --- | --- | --- | --- |
|  | Zn^2+^ | Mg^2+^ (sp) | Mg^2+^ (opt) | Na^+^ (sp) | Na^+^ (opt) | 2+ charge (sp) |
| ΔE^‡^ | 13.0 | 16.4 | 15.5 | 9.3 | 16.2 | 2.7 |
| ΔE | -5.1 | 1.5 | -0.4 | -14.6 | -2.3 | -9.0 |

# S9- Comparison between MCR and Alkaline Phosphatase

The MCR active site is structurally similar to that of the classical alkaline phosphatases (AP). Figure S2 shows a structural alignment of the two enzymes based on the relative positions of the respective phosphoryl acceptor residues (Thr285 in MCR-1 and Ser102 in AP, PDB 3TG0^1^) and Zn^2+^ ions. Notably, the two enzymes share very similar first coordination shells for Zn1 (MCR), and similarly positioned Zn^2+^ ions and bound phosphate moieties. However, although the inorganic phosphate in AP occupies the same position as the phosphate in phosphorylated MCR-1, the AP phosphate is in an inverted configuration. Furthermore, while the Zn1-bound Asp51 of AP, which is structurally equivalent to MCR-1 Glu246, makes similar mono-dentate coordination of Zn1, in AP the unbound oxygen atom of Asp51 coordinates a Mg^2+^ ion. This Mg^2+^ ion is absent from MCR-1, and the unbound oxygen of Glu246 instead forms a hydrogen bond with the side chain of Asn329. This suggests that, in MCR enzymes, Asn329 can substitute for the AP Mg^2+^ ion and position and orient the Glu246 carboxylate to activate the Thr285 nucleophile. Thus, provision of a positively charged group in this region of the active site can apparently stabilise the general base (Asp51 or Glu246) involved in nucleophile activation. Hence, despite their structural similarity, AP and MCR differ mechanistically, with MCR not requiring an additional cation for nucleophile activation, and, according to our findings, utilising only a single Zn^2+^ ion for phosphoryl transfer to Thr285.

|  |
| --- |
| Figure S2. MCR Enzymes Possess an Alkaline-Phosphatase-like Active Site. Structural alignment of MCR (PDB 5LRN, Zn2 of 5LRM) and Alkaline Phosphatase (PDB 3TG0^1^) based on the positions of first coordination shell of Zn1 in MCR. |

# Coordinates

Cartesian coordinates of the optimized geometries of systems shown in Tables S1 and S3 are attached to this supporting information in XYZ format.

# References

1. P. Hinchliffe, Q. E. Yang, E. Portal, T. Young, H. Li, C. L. Tooke, M. J. Carvalho, N. G. Paterson, J. Brem, P. R. Niumsup, U. Tansawai, L. Lei, M. Li, Z. Shen, Y. Wang, C. J. Schofield, A. J. Mulholland, J. Shen, N. Fey, T. R. Walsh and J. Spencer, *Sci. Rep.* 2017, **7**, 39392.

2. Gaussian 09, Revision A.02, M. J. Frisch, G. W. Trucks, H. B. Schlegel, G. E. Scuseria, M. A. Robb, J. R. Cheeseman, G. Scalmani, V. Barone, B. Mennucci, G. A. Petersson, H. Nakatsuji, M. Caricato, X. Li, H. P. Hratchian, A. F. Izmaylov, J. Bloino, G. Zheng, J. L. Sonnenberg, M. Hada, M. Ehara, K. Toyota, R. Fukuda, J. Hasegawa, M. Ishida, T. Nakajima, Y. Honda, O. Kitao, H. Nakai, T. Vreven, J. A. Montgomery, J. E. Peralta, F. Ogliaro, M. Bearpark, J. J. Heyd, E. Brothers, K. N. Kudin, V. N. Staroverov, R. Kobayashi, J. Normand, K. Raghavachari, A. Rendell, J. C. Burant, S. S. Iyengar, J. Tomasi, M. Cossi, N. Rega, J. M. Millam, M. Klene, J. E. Knox, J. B. Cross, V. Bakken, C. Adamo, J. Jaramillo, R. Gomperts, R. E. Stratmann, O. Yazyev, A. J. Austin, R. Cammi, C. Pomelli, J. W. Ochterski, R. L. Martin, K. Morokuma, V. G. Zakrzewski, G. A. Voth, P. Salvador, J. J. Dannenberg, S. Dapprich, A. D. Daniels, Farkas, J. B. Foresman, J. V. Ortiz, J. Cioslowski and D. J. Fox, Gaussian, Inc., Wallingford CT, 2016.

3. S. Grimme, S. Ehrlich and L. Goerigk, 2011, **32**, 1456-1465.

4. V. Barone and M. Cossi, *J. Phys. Chem. A*, 1998, **102**, 1995-2001.

5. F. Himo, *J. Am. Chem. Soc.*, 2017, **139**, 6780-6786.

6. M. R. A. Blomberg, T. Borowski, F. Himo, R.-Z. Liao and P. E. M. Siegbahn, *Chem. Rev.*, 2014, **114**, 3601-3658.

7. F. Neese, *WIREs Comput. Mol. Sci.*, 2012, **2**, 73-78.

8. F. Neese, *WIREs Comput. Mol. Sci.*, 2018, **8**, e1327.

9. S. F. Altschul, W. Gish, W. Miller, E. W. Myers and D. J. Lipman, *J. Mol. Biol.*, 1990, **215**, 403-410.

10. H. Berman, K. Henrick and H. Nakamura, *Nat. Struct. Mol. Biol*, 2003, **10**, 980.

11. A. Anandan, G. L. Evans, K. Condic-Jurkic, M. L. O’Mara, C. M. John, N. J. Phillips, G. A. Jarvis, S. S. Wills, K. A. Stubbs, I. Moraes, C. M. Kahler and A. Vrielink, *Proc. Natl. Acad. Sci. U.S.A,* 2017, **114**, 2218-2223.

12. M. A. Lomize, I. D. Pogozheva, H. Joo, H. I. Mosberg and A. L. Lomize, *Nucleic Acids Res.*, 2012, **40**, D370-D376.

13. R.-Z. Liao, J.-G. Yu and F. Himo, *J. Chem. Theory Comput.*, 2011, **7**, 1494-1501.
