# Supplementary figures and images for: Catalytic mechanism of the colistin resistance protein MCR-1
Source: Org Biomol Chem. 2021 Feb 16;19(17):3813–9. doi: 10.1039/d0ob02566f (PMC8097703; doi:10.1039/d0ob02566f)

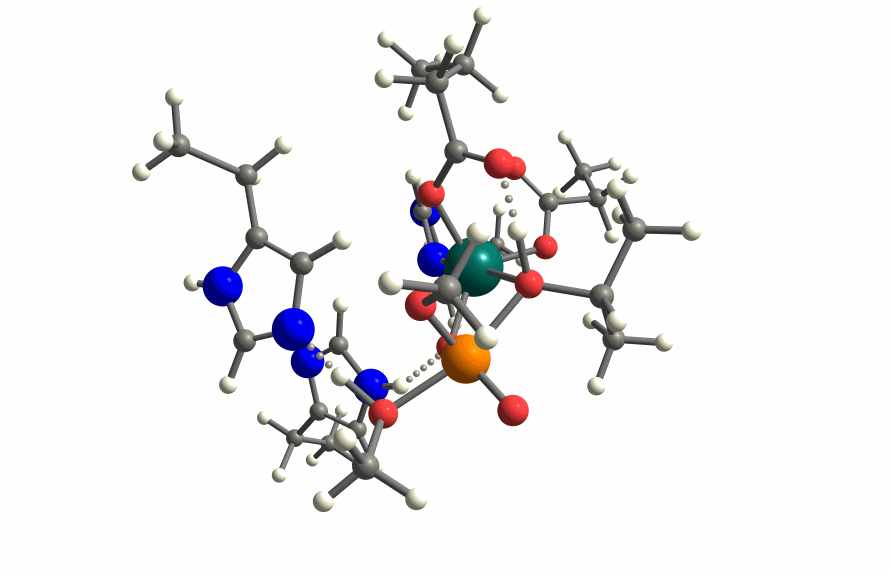

Supplement: OB-019-D0OB02566F-s003 [file OB-019-D0OB02566F-s003.gif]
